# Suspension feeders: diversity, principles of particle separation and biomimetic potential

**DOI:** 10.1098/rsif.2021.0741

**Published:** 2022-01-26

**Authors:** Leandra Hamann, Alexander Blanke

**Affiliations:** Institute of Evolutionary Biology and Animal Ecology, University of Bonn, An der Immenburg 1, 53121 Bonn, Germany

**Keywords:** filtration, particle separation, suspension feeder, suspension-feeding mechanism, biomimetics, bio-inspiration

## Abstract

Suspension feeders (SFs) evolved a high diversity of mechanisms, sometimes with remarkably convergent morphologies, to retain plankton, detritus and man-made particles with particle sizes ranging from less than 1 µm to several centimetres. Based on an extensive literature review, also including the physical and technical principles of solid–liquid separation, we developed a set of 18 ecological and technical parameters to review 35 taxa of suspension-feeding Metazoa covering the diversity of morphological and functional principles. This includes passive SFs, such as gorgonians or crinoids that use the ambient flow to encounter particles, and sponges, bivalves or baleen whales, which actively create a feeding current. Separation media can be flat or funnel-shaped, built externally such as the filter houses in larvaceans, or internally, like the pleated gills in bivalves. Most SFs feed in the intermediate flow region of Reynolds number 1–50 and have cleaning mechanisms that allow for continuous feeding. Comparison of structure–function patterns in SFs to current filtration technologies highlights potential solutions to common technical design challenges, such as mucus nets which increase particle adhesion in ascidians, vanes which reduce pressure losses in whale sharks and changing mesh sizes in the flamingo beak which allow quick adaptation to particle sizes.

## 1. Introduction

Suspension feeders (SFs) are a group of organisms with the common ability to separate food particles from suspension for nutrition [1,2], which includes organisms ranging from sponges to birds [3,4]. Since the late Tonian Period, 1000–720 Ma, SFs form habitats by mixing sediments, influencing particles fluxes, and moving high volumes of water [5,6]. Consequently, SFs altered light penetration depths, oxygenation levels and the distribution of dissolved organic carbon [7–9].

Suspension-feeding mechanisms (SFMs), which we define as all steps that enable separation of particles from the surrounding water, from the first encounter to the ingestion into the oesophagus, show a high diversity today. This diversity most likely resulted from niche partitioning, i.e. positive selection for the retention of certain particle size ranges from the heterogeneous seston [10,11]. Due to the high ratio of particle size to SF size, SFs provide small particles to higher trophic levels in aquatic ecosystems, e.g. products of primary production in the water column reach benthic habitats through the production of faecal pellets, subsidence of mucus and other biomass, and thus is an important linkage in the food web, known as benthic–pelagic coupling [12,13]. The diversity and species richness of SFs affect ecosystems because of their influence on plankton abundance, filtration rates and nutrient fluxes [14–16]. SFs also impact human living: Suspension-feeding herring, sardines and anchovies are relevant food sources [17] while bivalves and crustaceans are used as biofilters for water clarification [18–21].

Besides their ecological role, the separation mechanisms by which SFs separate food particles have also been of interest for engineers. The SFMs of manta rays inspired a nanofibrous membrane for oil–water separation [22] and led to the identification of a novel non-clogging filtration mechanism, called ricochet filtration [23], whereas suspension-feeding fish have inspired a helical, cross-step filter for collecting harmful algae [24].

Based on technical definitions [25], suspension-feeding processes are solid–liquid separations with particle recovery and the biological mechanisms show several similarities to technical ones. Natural and technical separation processes are divided into: (i) transport of the suspension to the separation medium, (ii) flow past the separation medium, (iii) separation of particles and (iv) particle removal from the separation medium.

Based on an extensive literature search, we developed a set of biological and technical parameters to systematically describe and classify SFMs and screened the animal kingdom for different SFMs.

## 2. A biomimetic approach to suspension feeders

The literature screening included scientific search portals (SCOPUS, Google Scholar) as well as biomimetic databases (www.asknature.org) up to December 2020 to identify as many SFs as possible and find SFMs that have not yet been considered in a biomimetic or technical context (electronic supplementary material, table S1). Because a detailed description of the SFM in each species would go beyond the scope of this review, species with a largely similar SFM within a taxonomic level (i.e. genus, family, order, class or phylum) were grouped and described briefly (electronic supplementary material, table S2). If sufficient data were available for one species to fully describe the SFM, it was chosen as a representative for the group, e.g. *Mytilus edulis* for all bivalves, otherwise the basic mechanism was described for the taxon, e.g. sponges. In the case of arthropods with their high diversity, only the groups with the best described SFMs were included.

**Figure 1.**
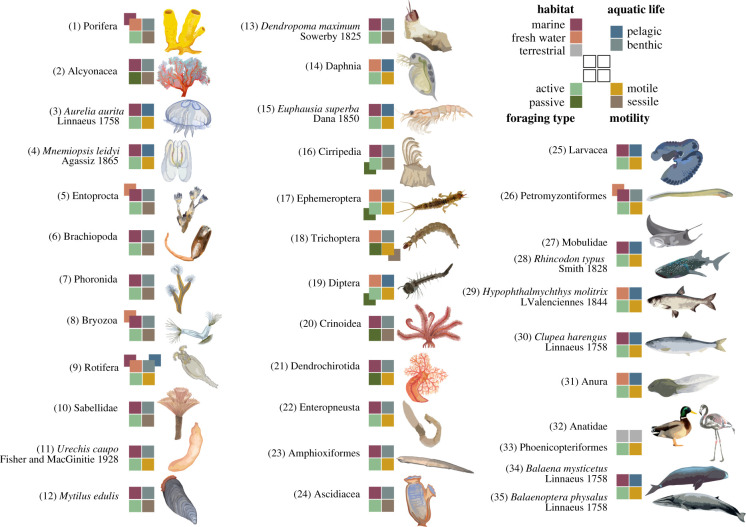
Overview of the selected SFs within the Metazoa with a focus on functional aspects. Each selected organism or organism group represents one SFM. Coloured squares indicate characteristics of biological parameters for each group: habitat (marine, freshwater, terrestrial), aquatic life (pelagic, benthic), foraging type (active, passive) and motility (motile, sessile). Numbering of each SF is consistent with table 1. A short description of each SFMs is in electronic supplementary material, table S2. For individual references, see electronic supplementary material, table S4.

Organisms were not considered for a detailed description if (i) they went extinct (e.g. pterosaurs), (ii) their feeding apparatuses have been mentioned only briefly thus far (e.g. sieve-like teeth in the crab-eater seal *Lobodon carcinophagus* [26]), (iii) they are mainly assigned to other feeding strategies (e.g. deposit-feeding cucumbers [27]), (iv) ciliary feeding larval stages [28] and (v) protists [29]. Filtration of molecules, such as in kidneys or aquaporins, was excluded as these mechanisms are not an aquatic feeding strategy [30]. Although not exhaustive regarding phylogenetic diversity, a total of 35 organisms and organism groups were selected (figure 1; electronic supplementary material, table S2) to cover the diversity of morphological principles, which we subsequently evaluate for their potential to inspire technical particle filters.

**Table 1.** The biological traits of each SFM are clustered and presented for each parameter (there is no relation between the columns). The numbers represent the SFs according to figure 1. Subunits in SFs, e.g. choanocyte in sponges, polyps in gorgonians, zooid in bryozoans, are indicated by (*). For individual references, see the numbers in electronic supplementary material, table S5.

**separation media**	**geometry**	flat (2, 14, 16, 19)	flat (in pipe) (12, 13)	funnel (1*, 2*, 7, 8*, 9, 10, 18, 20)	funnel (in pipe) (6, 11, 22, 23, 24, 26, 27, 28, 29, 30, 31, 32, 33, 34, 35)	others (1, 3, 4, 9, 15, 17, 21, 25)		
	**material of separation medium**	cell structures (1*)	epidermis (2, 3, 20, 21, 27, 28, 29, 30)	cilia (5, 6, 7, 8*, 9, 10, 12, 22, 23)	mucus (3, 4, 11, 13, 22, 23, 24, 25, 26, 31)	silk (18)	chitin (14, 15, 16, 17, 19)	horn/keratin (32, 33, 34, 35)
	**mesh design**	flat surface (3, 4)	first level of branching (1*, 2*, 3, 5, 9, 22, 32, 33, 34, 35)	second level of branching (2*, 6, 7, 8*, 10, 12, 14, 16, 17, 19, 27, 31)	third level of branching (14, 15, 20, 28, 30)	net (11, 13, 18, 23, 24, 25, 31)	‘spongy’/3D (1, 2, 21, 29)	
	**particle size**	<1 µm (1*, 11, 14, 19, 23, 25)	1–100 µm (1, 2, 4, 5, 6, 7, 8, 9, 10, 11, 12, 14, 15, 17, 18, 19, 20, 22, 23, 24, 25, 26, 27, 29, 31, 32)	100–1000 µm (1, 3, 20, 21, 25, 26, 27, 28, 30, 32, 33)	1–10 mm (21, 38, 32, 33, 34)	>10 mm (35)		
**separation type**		no filtration (2, 3, 4, 5, 7, 8, 9 10, 14, 19, 20, 21)						
		dead-end filtration (1, 7, 11, 12, 13, 15, 16, 17, 18, 22, 23, 24, 31, 32, 33, 35)						
		cross-flow filtration (7, 8, 25, 27, 28, 29, 30, 34)						
**fluid dynamics**	**driving force**	none (passive) (1, 2, 4, 13, 16, 17, 18, 19, 20, 21)	ciliary (+flagellar) movement (1*, 4, 5, 6, 7, 8*, 9, 10, 12, 22, 23, 24, 25)	movement of appendages (14, 15, 16, 17, 19, 25)	pumping (11, 22, 26, 28, 29, 31, 32, 33,)	forward movement (3, 27, 28, 30, 34, 35)		
	**water velocity (inflow)**	<0.1 cm s^−1^ (1, 5, 10, 12, 22)	0.1–1 cm s^−1^ (2*, 4, 7, 8*, 11, 14, 23, 24, 25)	1–10 cm s^−1^ (2, 3, 6, 15, 16, 17, 20, 25)	>10 cm s^−1^ (16, 18, 19, 27, 28, 30, 34, 35)			
	**flow regime (at mesh)**	creeping flow (Re <1) (1*, 2*, 8*, 9, 10, 12, 14, 15, 19, 22, 24,)	laminar flow (Re 1–50) (2, 3, 4, 6, 8*, 16, 17, 19, 20, 22, 25, 27, 28, 29, 30, 34, 35)	turbulent flow (Re >50) (27, 33, 34, 35)				
**cleaning**	**working mode**	continuous (1, 2, 3, 4, 6, 7, 8, 9, 10, 12, 14, 15, 16, 17, 19, 20, 21, 22, 23, 24, 25, 27, 28, 29, 30, 31, 32, 33, 34)	discontinuous (11, 13, 18, 25, 35)					
	**cleaning**	direct ingestion/phagocytosis (1, 5, 11, 13, 25)	ciliary transport (3, 4, 5, 6, 7, 8, 9, 10, 12, 20, 22, 31)	mucus (10, 12, 22, 23, 24, 26, 31)	feeding, scraping, combing off (2, 14, 15, 16, 17, 18, 19, 21, 32, 33, 35)	back flush (24, 28, 34)	non-clogging mechanism (25, 27, 28, 29, 30 34)	

The technical process of *filtration* is best comparable to SF. It describes a separation process using a filter medium to remove solid particles, microorganisms or droplets from a fluid [25]. Filters can remove particles from a fluid to receive a clean fluid (clarification), or they can retain valuable materials from a fluid (recovery) [31]. Based on these definitions, suspension-feeding processes are solid–liquid separations with particle recovery.

Because suspension-feeding, and especially filter-feeding, is similar to technical definitions of filtration, we propose the description of SFs using 12 technical parameters which are already established in particle separation processes such as particle properties, separation medium, fluid dynamics and cleaning of the separation medium (electronic supplementary material, tables S3 and S5) in addition to six ecological parameters (electronic supplementary material, tables S3 and S4) from previous biological descriptions (electronic supplementary material, table S6). Based on convergent SFMs, groups were clustered to each parameter (table 1) and corresponding literature presented for each SF (electronic supplementary material, tables S4 and S5). The groups also show the evolution of similar traits in response to the same boundary conditions that indicate structure–function relations and high biomimetic potential [27,28]. To account for the diversity of SFs that include typical filtration mechanisms but also other particle separation techniques, all technical terms including the term ‘filtration’ were changed to ‘separation’, i.e. ‘filter medium’ was changed to ‘separating medium’, ‘particle filtration’ to ‘particle separation’. In technical terms, the retained particle mass is called the retentate, the clean fluid that passes the filter is called filtrate [25].

## 3. Ecological description

SFs live in marine and aquatic environments with SF birds as the only solely terrestrial SFs dependent on aquatic environments (figure 1; electronic supplementary material, table S4). Insect larvae and tadpoles are the only groups that live exclusively in freshwater environments. Species within bryozoans, rotifers, bivalves, crustaceans, ammocoetes and fishes are present in freshwater and marine environments.

Benthic SFs are mainly sessile and live epifaunal on substrates or infaunal in burrows within the sediments such as ammocoetes [32]. Benthic SFs, such as the spoon worm *Urechis caupo*, enteropneusts, the sea snail *Dendropoma maxima* or lancelets are motile (or hemisessile) but remain stationary while feeding [33–36]. Through the building of substrates by tube-dwelling worms, bivalves or suspension-feeding corals, some SFs also act as ecosystem engineers influencing biogeochemical processes [2,7].

Habitat depth ranges from intertidal zones for barnacles and ascidians [37,38] down to the deep sea for sponges or brachiopods [39,40]. Pelagic SFs are motile by active swimming or drifting [41] and feed in varying depths, with whale sharks also feeding at the water surface [42] and suspension-feeding whales diving down several hundred metres [43,44]. Suspension-feeding usually is developed throughout the life or in adult life stages, but can also occur only in the larval stage such as in freshwater insects [45], anurans [46], lamprey larvae [47] or marine, invertebrate larvae [28]. Juvenile fish switch to filter-feeding at a species-specific size during growth [48].

Active SFs can influence local flow fields producing a feeding current by ciliary movement, pumping or forward motion [2,49] while passive SFs, such as gorgonians, crinoids or dendrochirotid sea cucumbers, retain particles from the ambient current [3,50].

## 4. Seston: the diverse food particles for suspension feeders

**Figure 2.**
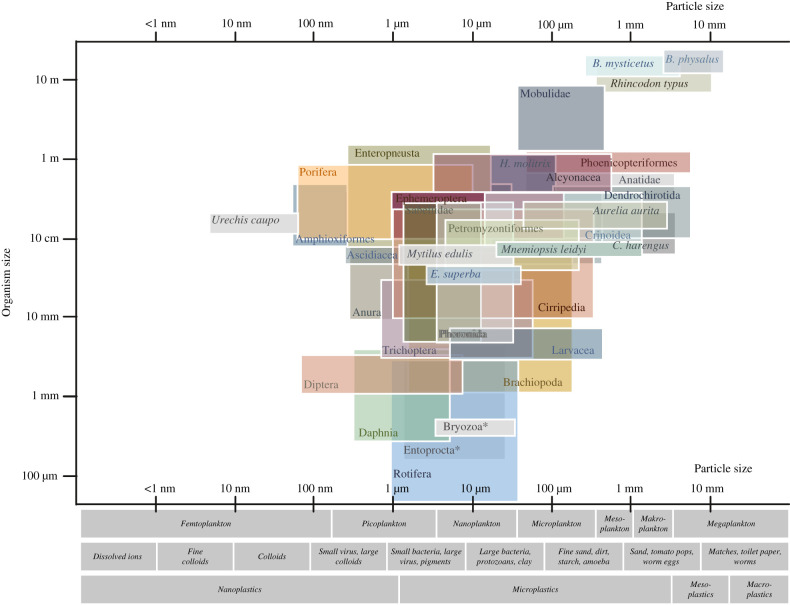
Size of the SFs (except *Dendropoma maximum*) and particle size of seston. Each box indicates the range of organism size and food particle size. For individual references, see electronic supplementary material, table S4. The colours are only used for visual reasons. Examples of seston particles are listed under the *x*-axis and compared to typical particle sizes found in waste water treatment and microplastics. For individual references of particle sizes, see electronic supplementary material, table S7.

SFs feed on seston, which includes all particles suspended in water regardless of their nature and origin, and mainly consists of plankton and detritus [15,51]. Plankton is commonly categorized by size (figure 2; electronic supplementary material, table S7) with the smallest size fraction consisting of viruses, followed by bacteria and protists. Protists range from 1 µm (flagellates) up to 1 cm (foraminifera) while phytoplankton ranges mainly between 2 µm and 200 µm. Macro- and mega plankton consists of invertebrate to vertebrate zooplankton including their life stages, among them are also SFs such as crustaceans [52]. Detritus and non-living matter ranges from dissolved or colloidal organic matter up to dead organic matter or marine snow several millimetres in size (figure 2; electronic supplementary material, table S7).

SFs do not seize individual prey but feed on a range of particle sizes (figure 2). Despite the relatively small size of seston, the ability to harvest small food particles in large amounts allows SFs to grow large with a particle to body length relation of about 1 : 10^2^ to 1 : 10^4^ [53,54]. SFs range from less than a millimetre (rotifers and bryozoans) up to 30 m for baleen whales and there is a positive correlation between SF size and food size (figure 2).

Small SFs, such as insect larvae, retain particle sizes down to colloidal particles [45], the spoon worm *U. caupo* can feed on 4 nm particles [55], corals and ascidians feed on bacteria [56], while larvaceans or bivalves retain viruses [57,58]. SFs feed on particles at least over two orders of magnitude in size, most of them in the range of 1–100 µm (figure 2). Data on preferred particle sizes are scarce for particles below 1 µm, which could be due to methodological detection difficulties [59].

SFs cope with varying seston concentrations and availability, which depend on habitat and local and seasonal dynamics [60,61]. Standing stocks of phytoplankton were calculated between 1 µg and 100 µm l^−1^ of oceanic waters, 5 µg and 1700 µg l^−1^ of coastal waters and 7 µg and 6800 µg l^−1^ of inshore waters [1]. Vertical migration of plankton changes daily seston concentrations in local areas and leads to behavioural changes in pelagic SFs, such as larvaceans [62], herrings [63], suspension-feeding sharks [42,64], bowhead or rorqual whales [43,65], the latter feeding at sites with prey concentrations up to 10^5^ per m^−^^3^, equivalent to around 170 g m^−3^ [66]. Several benthic SFs can change their feeding behaviour [67–69] or switch to other feeding strategies such as deposit feeding depending on particle flux and concentration [34,70,71].

Although being predominantly non-selective, particle selectivity can be determined by physical constraints. A lower limit of particle size are mesh size or the physics of particle encounter, i.e. hydrosol filtration [72,73]. An upper limit for particle size is the opening of incurrent canals, such as in sponges [74], tunicates [53,69] and ammocoetes [47]. Some SFs such as bivalves can actively select particles: the opening size of the inflow siphon regulates pre-capture while mucociliary transport in the four gut areas allows for post-capture selectivity before digestion [75]. Similar to bivalves, brachiopods produce pseudofaeces with rejected particles [76]. Suspension-feeding ducks select particle sizes by the beak opening, thereby changing mesh size [77]. SFs that use mucus to increase adhesive forces might select particles based on their chemical composition [78,79]. Particles are retained on surfaces when adhesive forces are greater than the sum of drag and lift forces acting on the particle to remove it [73]. Other particle properties that might influence particle retention and selectivity are density, shape, chemical criteria or energy content [75,80,81]. Each SF is adapted to a specific particle size range optimum for which the retention efficiency and ingestion rate are highest [82,83].

## 5. Separation medium

**Figure 3.**
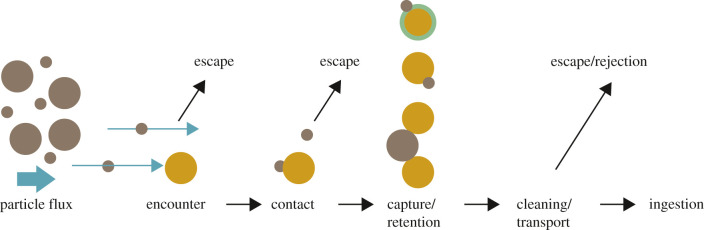
Steps of a generalized suspension-feeding mechanism, from the first particle encounter to ingestion (inspired by Waggett [84]). Particles (brown) encounter the separation medium (yellow) in direction of flow (blue arrows). According to hydrosol filtration theory, particles encounter the separation medium based on at least one of five mechanisms: (i) direct interception, (ii) inertial impaction, (iii) gravitational deposition, (iv) diffusion or motile-particle deposition and (v) electrostatic attraction [72,85]. After contact, particles can be captured through sieving or adhesion, e.g. through mucus (green). Particles can escape from the separation medium at each step or be actively rejected by some SFs during cleaning and before ingestion. Ingestion is the point of entry of particles into the oesophagus.

The separation medium is usually permeable and serves as a barrier to components in the suspension [31]. Geometry, physical dimensions and the separation medium's chemical properties influence water flow and particle retention in SFs (figure 3).

### 5.1. Geometry

**Figure 4.**
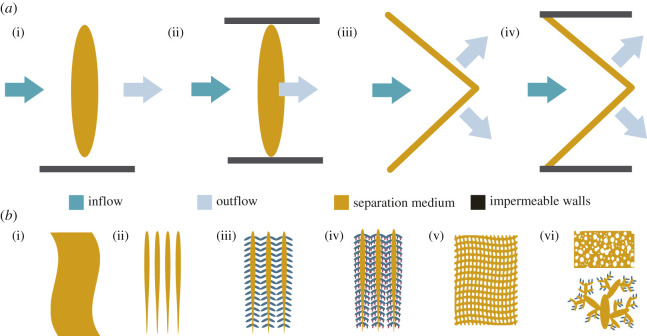
(*a*) Geometry of the separation medium (yellow) can be (i) flat and open, (ii) flat and enclosed, (iii) funnel-shaped and open or (iv) funnel-shaped and enclosed. Walls (grey) show if the separation medium is open or enclosed. Direction of flow is indicated by blue arrows. (*b*) Design of separation media to create surfaces, meshes and pores: (i) flat, (ii) first level of branching, (iii) second level of branching, (iv) third level of branching, (v) net structure and (vi) higher branching and porous media.

The separation medium is formed by body parts, such as appendages, inner structures like the pharyngeal basket, the body or external structures like excreted mucus nets (table 1; electronic supplementary material, table S5). The geometry of separation media has been described as funnel-shaped [86,87] or flat [88]. It can be extended in the open water stream or enclosed by the SF's body, burrows or other sorts of casing. This differentiation is not trivial because, in technical terms, a filter is a device that typically holds the separation medium across the fluid in such a way that all the fluid has to pass the separation medium [31]. Thus, we suggest that only enclosed separation media in SFs are filters (figure 4*a*), and, hence, filter-feeding is a particular case of suspension-feeding [2].

Separation media in SFs can be described by geometry and the open or enclosed position (figure 4*a*). The calcareous or gorgonin-based skeletons of gorgonians grow perpendicular to the fluid flow and they are an example for an open and flat separation medium [89]. Water flows through or around the space between the skeleton branches and particles are caught with the tentacles of the polyps. Suspension-feeding arthropods sweep their flat feeding appendages through the water [90–92]. The setules on the appendages in daphnids [93] and the gill of the bivalve *M. edulis* [94] can be angled, similar to pleated filter media used in common technical filters [95]. This provides elasticity for the filtering apparatus, increases the filtering area and decreases flow velocity at the mesh [88,93]. The marine snail *D. maxima* builds meshes across the opening of its burrow to retain particles [33].

The microvilli of choanocytes in sponges and the lophophores in entoprocts, bryozoans and phoronids are in the shape of open funnels (table 1; electronic supplementary material, table S5). The gill crown of sabellid worms extends as a spiralling funnel [96]. The arms of crinoids form a funnel and can be actively directed into the current [97]. The nets of trichopteran larvae are also funnel-shaped [98].

Deuterostomes such as hemichordates, cephalochordates, ascidians, ammocoetes, mobulid rays, tadpoles and species such as whale shark, silver carp, herring, fin whale or the bowhead whale, have funnel-shaped separation media within the pharynx (table 1; electronic supplementary material, table S5). The worm *U. caupo* builds a funnel-shaped net in its burrows [36]. The enclosed lophophore in brachiopods can vary in shape but is often funnel-shaped [99]. Ctenophores, sponges, the moon jelly *Aurelia aurita*, rotifers and mayfly larvae show other geometries of separation media, which do not fit the above classification. Examples for even more complex geometries are the highly branched arms of dendrochirotid sea cucumbers, the external filter house of larvaceans and the filtering basket formed by the legs of Antarctic krill (table 1; electronic supplementary material, table S5).

The total area of the separation medium exposed to the on-streaming fluid is called the *separation area* or *effective separation area* (effective filtration area in technical terms [31]). In gorgonians, the effective area is nearly equal to the skeleton area and therefore correlates with organism size [100]. By adding up all areas of the filtering pads in whale sharks, the filter area measures 10–12 m^2^ in a 6 m individual [42]. However, the effective area can change dynamically, especially in changing flow fields: in sea lilies, the area decreases with increasing flow because the pinnules bend backwards with higher drag force [101]. The filter basket of Antarctic krill can be actively expanded and compressed by the organism to pump water through it [102].

### 5.2. Tissues and materials

Different tissues and materials influence particle capture in SFs (table 1; electronic supplementary material, table S5). The flagellum of the choanocytes in sponges creates a current towards the microvilli and the cell body, where particles are taken up by the cell through phagocytosis [103]. Epithelia and the epidermis of the tentacles of gorgonians and the moon jelly *A. aurita*, the tube feed in crinoids, and the tentacle arms of dendrochirotid sea cucumbers have first contact with particles. In suspension-feeding Chondrichthyes, like mobulid rays and whale sharks, the separation medium consists of filter plates between the gill arches [104]. In bony fish, the gill arches are equipped with gill rakers (fuzed in silver carp), which build a screen for the water flowing through the mouth, towards the gills and out under the operculum [105].

Small SFs such as rotifers, entoprocts, lophophore-bearing brachiopods, bryozoans and phoronids predominantly use cilia to catch and retain particles [106]. Larger SFs with cilia are sabellid worms, the blue mussels *M. edulis*, enteropneusts and lancelets. Four different mechanisms of particle retention with cilia are distinguished based on the number of ciliary bands, the stiffness of the cilia and how the cilia move to interact with the particles: upstream collecting, ciliary sieving, cirri trapping and downstream collecting [107]. In the bivalve *M. edulis* and phoronids, different types of cilia are involved. While the lateral cilia create a current and trap the particles, the frontal cilia transport the particles towards the gut [106,108]. Bryozoans and rotifers can control the water current by cilia in such a way that particles are directly driven towards the mouth [109].

In various taxa, mucus is involved in suspension-feeding during retention, cleaning or transportation of the particles. Mucus used in separation media can be divided into three categories. (i) The ctenophore *Mnemiopsis leidyi*, the jelly fish *A. aurita* and dendrochirotid sea cucumbers cover surfaces to increase particle adhesion [71,110]. (ii) Lancelets, ascidians, ammocoetes and tadpoles have internal mucus nets. These nets are supported by structures, such as the pharyngeal basket, and transported with cilia. In ascidians, a continuously secreted mucus net covers the pharyngeal basket, which retains particles down to 1 µm and allows the water to pass the filter at low resistance [111]. (iii) The spoon worm *U. caupo* and the sea snail *D. maxima* build mucus nets externally within their burrows to catch particles and ingest the particle-laden mucus periodically [33,36]. Larvaceans secrete a complex filter structure around them, which is several times larger than the organism and consists of a coarse-meshed outer house and a fine-meshed inner house to concentrate the food particles towards the mouth [112].

Even though mucus has been recognized early as a relevant part of suspension-feeding [113], its physical and chemical properties are not well understood compared to the information available for terrestrial organisms using mucus [114]. Generally, mucus is highly viscous and resembles an elastic gel with a high adsorption potential for particles. When particles touch a mucous surface, the mucus will engulf the particles and thereby retain them [114]. The spoon worm *U. caupo* [55] and larvaceans [78] retain particles down to 4 nm through the adhesive forces of mucus. Mucus properties, such as its electrical charging, can influence particle retention [115]. In SFs, the production of the mucus net and its physical and chemical properties were studied for ascidians [116], larvaceans [53], the blue mussel *M. edulis* [117,118] and salps [119].

Caddisfly larvae build nets with silk strands in rivers and streams to catch particles from the passing current [45]. A viscous liquid is drawn through a fine orifice to produce the protein fibres [120]. Each larva can secrete up to 70 strands simultaneously. The diameter of the strands in different species can vary between 0.34 µm and 47 µm [121]. Caddisfly silk can double in length before it breaks so that the larvae are able to build their nets between rocks and stones in flowing waters, where the silk needs to withstand fluctuations of flow velocity and impacts of larger particles [120].

Chitin is the material for separation media in suspension-feeding crustaceans and insects. Daphnids, the Antarctic krill *Euphausia superba*, barnacles and mayfly larvae use legs with bristle-like setae on them to retain particles, while dipterans have bristle-like mouthparts (electronic supplementary material, table S5).

Compared to other keratinous structures, the α-keratin in whale baleen hanging as bristles from the upper jaw has a higher degree of calcification, which increases abrasion resistance, enhances fraying into bristles and increases strength and flexibility [123,124]. Keratinized structures also form fine lamellae at the rim of the upper and lower beak in suspension-feeding birds [125,126].

### 5.3. Media design and meshes

The design of separation media ranges from flat surfaces, over different degrees of branching and net-forming screen-like meshes, to spongy and highly branched structures forming porous media to create surfaces, meshes and pores (figure 4*b*).

Ctenophores and the moon jelly *A. aurita* have flat, prey-capturing surfaces. Because the separation medium has no meshes, the water does not flow through the separation medium but around it. Particles that encounter the surface are retained with adhesive surfaces, sometimes covered with mucus [110,127].

Several levels of branching form apertures, so water can flow through the separation medium (figure 4*b*). The microvilli of choanocytes in sponges, the tentacles of the moon jelly *A. aurita*, the cilia of lophophores or the lamellae in the beaks of ducks and flamingos are examples of the first level of branching (table 1; electronic supplementary material, table S5). Barnacles, where setae are equipped with smaller setulae [92], or mobulid rays, where the filtering lobes divide up into smaller structures [104], are examples for the second level of branching. Atlantic krill, where the primary setae have secondary setae with even smaller tertiary setae on them [102], have a third level of branching. In herrings, the gill arches have gill rakers which themselves have denticles to create almost rectangular meshes [128]. By contrast, whale sharks have the same level of branching, but irregular-sized meshes [42].

Different to branching structures that develop through growth processes, nets are formed by, for example, spinning processes [120]. The larvae of caddisflies can build food-capturing nets in flowing waters and act as a trade-off between processing large volumes of water and the water pressure [129]. The net design ranges from being elongated, sac-like, disc-like or branching into tubes. Nets tend to be larger in faster flows, and mesh sizes can be altered by the organism for the retention of specific particle sizes [130,131]. Other net spinning and mucus-secreting SFs are the spoon worm *U. caupo*, the sea snail *D. maxima*, lancelets, ascidians, larvaceans and tadpoles (table 1; electronic supplementary material, table S5).

In engineering, the retention of particles on a two-dimensional mesh is called surface filtration, while the retention within a three-dimensional structure, i.e. pore, is distinguished as depth filtration or deep bed filtration [25,31]. In sponges, the water streams into the channels, where particles are retained by archaeocytes on the sides in addition to the particle retention by choanocytes in the filtering chambers [132]. During the ontogeny of silver carps, the gill rakers fuse and form a porous medium [133]. The highly branched skeleton of gorgonians and the arms of dendrochirotid sea cucumbers form a three-dimensional structure in the open flow [71,134].

### 5.4. Mesh size and particle size

Mesh size is the size of apertures in a screen or mesh [31] or the distance between structures that retain particles. The distance between the cilia in the entoproct lophophore is around 0.1 µm [135], the smallest mesh size of ascidian mucus nets is 0.2 µm by 0.5 µm [69] and the mesh sizes in mobulid rays range from 0.27 mm^2^ to 3.34 mm^2^ depending on species [136]. The ratio of apertures to effective area is called the open area ratio, the ratio of pore volume to total volume in porous separation media is the porosity [31]. The open area ratio of the feeding appendages in daphnids ranges from 0.5 to 0.7 [137]; it is 0.46–0.6 in whale sharks [42] and the porosity in mucus nets in ascidians is 90–98% [69].

Mesh or pore size can be changed by passive forces, e.g. the distance between baleen fringes changes with the flow velocity [44,138]. Depending on the food source, flamingos and ducks can alternate the distance between the upper and lower jaw and hence adjust the mesh size actively between the upper and lower lamellae [125]. Because of these dynamic changes of the mesh or pore size, retention mechanisms other than sieving [72] and the fact that not all physical properties are known, it is not possible to predict the size of particles that are retained by a specific SFM, as is the case for technical filters by the cut-off point [31]. Thus, we suggest that the particle sizes, which have been ingested by SFs, are a better indicator for the sizes which can be retained by the SFMs (figure 2).

## 6. Fluid dynamics

In nature, the fluid of suspension-feeding is water, but in technical filters other liquids or even gases are treated in separation processes [31]. Flow velocity and flow regime of the fluid play a major role in particle motion towards the separation medium and the final encounter with the separation medium (figure 3) [3,4].

### 6.1. Type of separation

SFs are distinguished into filter feeders (FFs), which have a filter comparable to technical designs where all fluid has to pass the filter medium, and non-FFs [2]. Based on the direction of flow, dead-end and cross-flow filtration can occur. In dead-end filtration, the fluid flows orthogonally towards and through the filter medium; in cross-flow filtration, the flow streams tangentially along the separation medium [25,31]. Cross-flow filtration is present in SFs, such as in the external filter houses of larvaceans, and the internal SFMs in mobulid rays, whale sharks, suspension-feeding fish, such as silver carps and herrings, and bowhead whales (table 1; electronic supplementary material, table S5). The tangential flow pushes the particles across the surface of the filter medium towards the oesophagus. Thus, particles are constantly removed and increased in concentration by fluid flow before being swallowed or ingested [112,139].

Brachiopods, the spoon worm *U. caupo*, the blue mussel *M. edulis*, the sea snail *D. maxima*, the Antarctic krill *E. superba*, barnacles, ephemeropterans, trichopterans, enteropneusts, lancelets, ascidians, tadpoles, flamingos and Anatidae have enclosed separation media, in which particles are deposited upstream in the dead-end filter (table 1; electronic supplementary material, table S5). The flow around the separation medium, and its geometry, distinguishes dead-end from cross-flow FFs. In entoprocts, the separation medium is funnel-shaped, but the flow is very slow and not tangential [34]. Daphnids form a flat mesh with the setulae on the feeding appendages, but the flow streams across it instead of through it [140].

Ctenophores, gorgonians, moon jellies, rotifers, entoprocts, bryozoans, phoronids, sabellid worms, larvae of dipterans, crinoids, dendrochirotid sea cucumbers and daphnids are non-FFs (table 1; electronic supplementary material, table S5) because their separation medium is not enclosed, and the fluid can stream around it. However, the separation medium might still form meshes or pores to retain particles by sieving.

### 6.2. Driving force

Passive, benthic SFs often grow large, are stalked, or extend away from the sediments and the benthic boundary layer to reach into faster flows and collect particles with higher energetic content [85,141]. The caddisfly larvae of Macronema, which build their nets within tubes, use the pressure difference of incurrent and excurrent openings to drive the fluid through the net within the tube, a similar mechanism to a pitot tube [142].

Within technological applications, suspensions are transported by hydraulic pumps, vacuums or gravity towards and through the filter [25,31]. The ‘pumps’ of SFs are ciliary and flagellar movement, movement of appendages, oscillatory pumping and forward motion.

Small SFs, which use cilia to catch particles, often induce a feeding current with their cilia. This includes rotifers, entoprocts, brachiopods, bryozoans, phoronids, sabellid, bivalves, enteropneusts and lancelets (table 1; electronic supplementary material, table S5). The activity of cilia to induce a flow is also referred to as ciliary pump [143]. Despite the small size of the flagella of the choanocytes, sponges can induce relatively fast flows. The area of the flagellated chamber with the choanocytes is around 6000 times greater than the profile area of the excurrent canal. Thus, the flow velocity is multiple times slower around the flagella and increases in speed up to 0.2 m s^−1^ with decreasing area of the excurrent canals [142].

Suspension-feeding crustaceans and insect larvae sweep their feeding appendages through the water [1]. The feeding behaviour of barnacles is influenced by ambient flow conditions and can change direction and between active and passive [144]. In slow currents, barnacles actively move the feeding cirri through the water, while in fast currents, the cirri are held up because flow velocity is high enough to be filtered passively and thus save energy. To be able to extend the cirri in fast water currents, the cirri are mechanically robust to withstand the pressure without buckling or bending [92,145]. Larvaceans move their tail to pump water through their external filter houses [112].

Rhythmic contractions of the pharyngeal wall in lamprey larvae or buccal pumping in tadpoles induce a flow into the buccal cavity [4]. Flamingos open and close their bill while their tongue moves like a piston to suck in the water at the tip of the beak and expel it at the sides [125]. Lamprey larvae pump against sediment resistance and obtain suspended food particles from the water above and within the sediments [32].

Ram feeders, such as suspension-feeding fishes, whale sharks and bowhead whales, feed while swimming and take advantage of the forward motion to stream water towards their separation media [4]. Because their separation medium lies within the oral cavity, ram feeders might benefit from the continuity effect and the pressure drop to reinforce flow through the mouth, as shown for bowhead whales [146]. Ram feeding is characterized by a unidirectional flow, as opposed to bidirectional flow, e.g. in fin whales [4]. Fin whales accelerate and open their mouth fully to engulf their prey with a big gulp. Water flow is then reversed through the baleen plates, where particles are retained [147].

### 6.3. Flow velocity and pressure difference

Sponges, rotifers, sabellid worms, blue mussels and enteropneusts induce flow velocities smaller than 0.1 cm s^−1^. Ctenophores, gorgonians, bryozoans, phoronids, the spoon worm *U. caupo*, daphnids, lancelets and ascidians stream water between 0.1 cm s^−1^ and gorgonians, the moon jellies *A. aurita*, brachiopods, the Antarctic krill *E. superba*, barnacles, Ephemeropterans and crinoids induce flows between 1 cm s^−1^ and 10 cm s^−1^. Ammocoetes, larvae of trichopterans and dipterans, mobulid rays, whale sharks, herrings, bowhead whales and fin whales induce flows higher than 10 cm s^−1^ (table 1; electronic supplementary material, table S5).

Flow velocity can change depending on individual organism size or spatial and temporal conditions. In larvaceans, the flow velocity varied between 0.37 and 12.2 mm s^−1^ within 23 measured individuals with an allometric exponent of trunk length to the power of 2.5 [148]. The flow velocity in sponges is 0.009 mm s^−1^ at the collar slit of the choanocytes while being 2.9 mm s^−1^ in large excurrent canals of the same species [149]. Passive SFs in ambient, oscillatory flows are exposed to flow velocities ranging from no flow up to 15 cm s^−1^ [67,92].

The separation medium in the fluid flow creates resistance, a drag, which is expressed as the pressure difference across the separation medium [25]. It depends on the specific resistance of the separation medium and the fluid velocity: the higher the flow rate per unit area, the higher the pressure difference. A whale shark swimming at 1.1 m s^−1^ creates a pressure difference of 113 Pa at the filtering plates [42]. Bowhead whales induce a pressure difference between 1200 Pa and 4000 Pa depending on swimming velocity [146]. The driving force and thus flow velocity must be high enough to move fluid towards the separation medium and overcome the pressure drag [85].

### 6.4. Flow regime

The flow regime describes the flow structure and is expressed by the dimensionless Reynolds number (Re), i.e. the relation of inertial to viscous forces within a fluid [31,142]. In low Reynolds numbers of less than 0.1 (sometimes Re < 1 [150]), the flow is creeping, viscous forces dominate and the streamlines are parallel around a body [72]. With higher Reynolds number, inertial forces become more relevant, and the flow regime changes from laminar to turbulent [150].

The flow velocity and the characteristic length of particles or separation medium structures influence the local flow regime [150–152]. In most SFs, the characteristic length of the feeding element varies between 0.1 µm and 1 mm, and the flow regime is in the intermediate flow region between Re 0.5 and 50, where inertial forces are almost equal to viscous forces and streamlines begin to compress around a body [3,85,151,153,154]. Numerical models of the particle encounter in this flow regime show that with increasing particle radius and increasing Reynolds number at the collector, the encounter rates increase nonlinearly [73,151]. Within the pharynx in enteropneusts [34], between the lobes of ctenophores [155], and at the lophophore in brachiopods [156] the flow regime is around Re 1, i.e. inertial forces equal viscous forces.

Creeping flow at the separation medium has been calculated as Re 5.6 × 10^−4^ down to 6.09 × 10^−5^ for single cilia in rotifers [157], Re 0.00057 around choanocytes in sponges [103], Re 0.2 and lower at the ciliary bands of sabellid worms [158] and Re 0.0002 at the cilia in blue mussels [159]. These low Reynolds numbers indicate that particles are not removed through sieving because viscous forces are dominant, and thus it is energetically too expensive [140]. Due to high shear forces at low Reynolds numbers, particles are often individually directed by cilia along path lines towards the mouth [85,157].

Higher Reynolds numbers indicate turbulent flow and the formation of eddies [142]. The Reynolds number at which laminar flow becomes turbulent in size classes relevant for SFs is at Re > 200 [85] or as high as Re > 1000 [150] and depends on environmental conditions and geometries [142,160]. In large pelagic SFs, Reynolds numbers at the mesh have been determined up to 300 for mobulid rays [136], whale sharks [42], bowhead whales and fin whales. Dissipating energy caused by turbulence increases the energetic costs of SFs [156]. Thus, even large SFs are likely to induce a laminar flow regime to reduce energetic costs. Vanes on the downstream side of the whale shark have been assumed to act as collimators to remove turbulent eddies larger than the grid size [42,85].

A vortex-based mechanism was identified in suspension-feeding fish [161] and is suspected to occur in bowhead whales [162], both being cross-flow FFs. Ducks have been suggested to use turbulence to induce cyclonic vortices that separate particles by density [126]. The jelly fish *A. aurita* creates vortices with Reynolds numbers changing between 0 and up to 150 during the power stroke, which brings particles towards the bell margin [110].

The ambient flow of SFs is typically turbulent due to wind, tides and currents [85,155]. For benthic SFs, an ambient turbulent flow regime leads to particle mixtures and fluid exchange in the benthic boundary layers [85], which increases the particle capture rate [163]. However, the Reynolds number for benthic bivalves and ascidians ranges between 8 and 520 at the inhalant siphon. Hence, flow is laminar when entering the organism [164]. In colonial SFs, such as bryozoans, the morphology and packing of single units influence the overall flow field to induce excurrent flows to vent the colony [163].

## 7. Cleaning of separation media

After particles are retained by the separation medium, the particles have to be removed to maintain the function of the separation process. In technology, this process is referred to as cleaning and can be further distinguished as continuous or discontinuous cleaning, depending on the mechanism and frequency. An increase of particles that become stuck in a pore or mesh leads to increased drag and higher energy expenditure [25,31]. Sponges directly take up particles by phagocytosis when encountering the microvilli or the cell surface of choanocytes, and particles are engulfed by pseudopodial extensions if at a distance of several micrometres from the cell [165]. The spoon worm *U. caupo* and the snail *D. maxima*, which build external mucus nets, periodically eat their nets along with the retained particles [33,36].

In ciliary SFs, alteration of ciliary movements and reversed strokes lead to transport towards the gut, such as in rotifers [109], brachiopods [76] or phoronids [106]. Tentacle flicking pushes single particles towards the mouth [106,166]. In moon jellies, ciliated grooves transport particles towards the gut after being caught by tentacles and nematocysts [110]. Crinoids catch particles with their tube feet and pinnules. By flicking of the pinnules, the particles are moved to the food grooves, where cilia transport the particles towards the mouth [97].

Moon jellies [1], ctenophores [84], sabellid worms [158] or blue mussels [108] are transporting particles by cilia in combination with mucus. The continuous mucus net in ascidians, which aligns the pharyngeal basket, is transported by cilia towards the oesophagus, where it is rolled up into a string and digested with the attached particles [69,167]. Enteropneusts, lancelets, Petromyzontiformes and tadpoles entrap particles with mucus within their pharyngeal basket or buccal cavity and swallow the aggregation afterwards (table 1, electronic supplementary material, table S5).

Particles can be fed off, scraped off or combed off the separation medium mechanically. During the retraction of polyps in gorgonians [168] and bending of the arms towards the mouth in dendrochirotid sea cucumber [71], particles are wiped off within the mouth and ingested. Suspension-feeding crustaceans and insect larvae use legs or mouthparts as cleaning brushes to swipe off particles and pass them towards the mouth [92,102,121,169]. The tongues of flamingos [170] and suspension-feeding ducks [171] are covered with spines sweeping off the particles from the lamellae on the inner sides of the beak and transport them towards the oesophagus. Lunge feeding whales, e.g. fin whales, also mainly use their tongue to remove captured prey from the baleen fringes combined with other mechanisms [172,173].

In FFs that use cross-flow filtration, the tangential flow constantly removes particles from the separation medium and increases particle concentration near the oesophagus opening [112,162]. Larvaceans, mobulid rays, whale sharks, silver carp, herrings and bowhead whales use this non-clogging mechanism (table 1, electronic supplementary material, table S5).

In response to environmental conditions, such as high particle concentrations, some SFs can switch between cleaning mechanisms or adapt their cleaning behaviour. Whale sharks [42] and bowhead whales [43] use a mechanism that is known as back-flushing or back-washing in filtration technologies. The flow is reversed backwards through the separation medium to clear plugged or clogged particles from the meshes [31]. Back-flushing interrupts the feeding process and is only used by SFs when their usual cleaning techniques are unsuccessful [42,69].

If undisturbed, most of the selected SFs feed continuously (30 of 35 SFs). Only a few organisms interrupt the feeding process because particles need to be removed from the separation medium, including all SFs that build external mucus and silk nets. While the spoon worm *U. caupo*, the sea snail *D. maxima* and trichopterans eat their nets along with the retained particles, larvaceans repel their nets (table 1; electronic supplementary material, table S5). All have to rebuild their nets afterwards. Fin whales feed discontinuously because they catch their food in big gulps [172,173].

## 8. Biomimetic potential

Most SFs use filtration, i.e. the separation medium is held into the fluid so that all fluid passes it, as the mechanism of separation. Similar to filtration technologies, the type of flow is dead-end or cross-flow filtration. However, while cross-flow filtration in industrial applications retains small particles in ultra- and nanofiltration [174], SFs such as mobulid rays, whale sharks or baleen whales also retain particles up to 10 mm with this mechanism [23,42,175]. These organisms use varying material properties and/or fluid flows within their cross-flow filtration to influence the interaction of particles and the separation medium. In the ricochet mechanism of manta rays, the particles bounce off the filtering lobes towards the oesophagus [23], in pump-feeding fishes, the gill arches induce the formation of vortices known as cross-step filtration [24] and in bowhead whales, the flow is diverted by the tongue and pressed along the baleen fringes that change in porosity depending on flow speed [175]. In all of these mechanisms, particles smaller than the mesh size are retained and the tangential flow reduces the clogging rate.

Centrifugal separations in technical applications, that separate particles based on rotating baskets or sedimentation, are not common in nature. Even though some SFs influence fluid flow specifically and create vortices, they mostly rely on particle–material interactions. Therefore, the chemical and physical properties of the separation media are specifically adapted to increase the chances of retention after encounter (figure 3). For example, the addition of surfactants to change the surface charge of the particles in feeding experiments led to a decrease in retention of small particles in daphnids [122], which shows that the material properties of chitin increase particle retention. Mucus as separation medium has evolved convergently in several taxa of the SFMs (13 of 35) and aids in particle retention and transport. Even though the filter materials used in technology are highly diverse and include natural and synthetic, organic and inorganic materials [31], to our knowledge, mucus-like filter media that use adhesive forces to retain particles are rarely used in solid–liquid filtration technologies. For example, a hydrogel was inspired by plant tissue to absorb uranium from seawater [176] and membrane surfaces were manufactured with super-hydrophobicity for bioinspired oil–water separation [22]. Filtration media in industry are manufactured independent of the filter housing, with woven fibres, perforated sheets or sintered metals as common filter designs [174]. Most of the SFs built their separation media from one or several body parts by branching or bristling (figure 4). Filter and filtration media are thus inseparable and sometimes multifunctional, thus providing stability, or aid in locomotion or gas exchange. The external filter house of larvaceans is built from mucus, which gives stability and acts as the separation medium itself. The geometry of the separation media ranges from allegedly simple surfaces to complex spinned three-dimensional geometries, but it is in most cases funnel-shaped (figure 4), which, we assume, is one of the more efficient ways of increasing the filtration area. The setules on the appendages in daphnids [93] and the gill of the bivalve *M. edulis* [94] can be angled, similar to pleated filter media used in common technical filters to increase the filtration area [95,96]. The combination of several functions and the construction of complex filters could be made possible in the future through additive manufacturing or spinning technologies [175,177]. However, parametric studies on filtration efficiency which determine the influence of geometries found in SFs have not been carried out to date.

SFs require energy to cover the metabolic costs of growth, reproduction and feeding, i.e. foraging, the formation of separation media and creating a feeding current [10,80,178–180]. Therefore, SFs evolved along several of these fitness gradients [85,181]. An elongate rectangular mesh design can save up to 18% of silk material and requires less spinning movement in trichopteran larvae [98]. Ascidians grow in the shape of a pitot tube which induces a passive flow that relieves their ciliary pumping activity [37]. Because the energetic costs of filtration are proportional to hydrodynamic resistance under a constant flow rate, whale sharks and manta rays have vanes to reduce the pressure difference at the separation medium [23,42]. These SFs are large and their structures to optimize flow could be used to improve large filters with high throughput, such as industrial and public waste water treatments plants.

In SFs and technical filters, the particles are usually retained upstream of the separation medium. Exeptions are sabellid worms, which use cilia to collect particles after the water has passed the filaments of the gill crown [96]. All SFs evolved SFMs together with an inherent cleaning mechanism to remove the retained particles from the separation medium. Clogged filter media increase pressure differences and energy expenditure, and also negatively affect filtration rates. Cleaning and transport of particles is often achieved by the structures of the separation medium such as cilia, mucus or cell surfaces but SFs also use combing or back-flushing. Non-clogging mechanisms are also combined with cross-flow filtration: the fluid flows tangentially towards the separation medium, the particles are constantly removed and directed towards the oesophagus. This is in contrast to technical filters in which a cake is formed on the filtration medium by the layers of retained particles [174]. Cake removal is a problem, for example, in filter presses where the filtration process has to be interrupted to remove the cake [182]. By contrast, the majority of SFs have cleaning mechanisms that allow a continuous working mode (table 1) and take up the particles for nutrition at the same time.

Because most SFs are non-selective within the particle size range their SFMs are adapted for, this leads to an uptake of a heterogenic particle mixture, including anthropogenic particles, such as carbon fibres [183], metals [184] and microplastics. Microplastic uptake was reported for sponges, gorgonians, jelly fish, rotifers, sabellid worms, blue mussels, daphnids, Antarctic krill, barnacles, mayfly larvae and caddisfly larvae, crinoids, dendrochirotid sea cucumbers, tunicates, whale sharks, suspension-feeding fishes, tadpoles, suspension-feeding birds such as prions, and baleen whales (electronic supplementary material, table S4). Secondary and tertiary waste water treatment plants only retain 88% and 90% of microplastics and the rest is released into the environment, where they accumulate [185,186]. Seston and plastic particles have similar dimensions (figure 2) so that SFs feeding in a similar size range might be suitable biological models for microplastics filtration. Additionally, SFs have mechanisms that are selective for specific particle properties such as shape, size or chemical properties, which might be useful for applications to extract specific particles from a heterogeneous mixture, such as microplastics from waste water. Generally, appropriate biological models for technical applications can be identified based on similar boundary conditions found through the parameters presented here (table 1). Subtle variations within similar SMFs, e.g. within the 10 000 species of sponges, could then inform parametric studies. In environmentally relevant applications such as the retention of microplastics, aspects of sustainable product development also should be considered at an early design stage [187,188].

Within this review, we studied traits individually, but evolution leads to trade-offs and development with phenotypic and/or phylogenetic constraints on multiple traits. Examples in SFs are the jet propulsion of the moon jelly *A. aurita* that propels the organism forward and also streams particles towards the separation media. In filter-feeding fishes or manta rays, the gill arches are modified for nutrition, but they also serve gas exchange. Comparison and transfer-of-principles from nature to technology need to consider such multifunctional constraints when taking SFMs out of their natural context [189].

The abstraction into numerical or physical models enables testing and verification of the biological principles outside the environmental context and allows a first check of transferability and scalability. Filtration technologies can work with vacuum, high pressures or steam, to which biological systems might not be applicable because they work at ambient temperature, are adapted to water, and low pressure differences. Drum filters can operate up to pressure differences of 10 bar (1 MPa). Whale sharks as one of the largest SFs induce a pressure difference of around 113 Pa (pressure head) at a swimming speed of 1.1 m s^−1^ [42]. These systems could inspire designs which work at lower pressures. Recent technical developments are hydrophilic membranes with capillary entry pressure to replace vacuum or filtrate pumps [182]. An example besides SFs are bioinspired membranes with embedded aquaporins developed for ultrafiltration [30].

Filtration technologies change depending on the scale due to physical or chemical restrictions. Coarse particles greater than 10 µm are retained by vacuum disc filters whereas small particles are retained by gas overpressure filtration. SFs range from several hundred micrometres to 25 m in size (figure 2) and also here the separation principles change. On smaller scales and Reynolds number up to 50, cilia and mucus are more common to retain small particles. Large ram-feeding fishes, sharks and whales use cross-flow filtration in which high amounts of particles are retained from water velocities higher than 10 cm s^−1^. SFs often have an optimal range were particle retention is close to 100% [82,83,190]. When an application of a bioinspired filter is outside its original scale, a check for scalability is necessary. Dimensionless numbers such as the Reynolds number offer a good approximation of fluid dynamical aspects [189].

Engineered solutions result from decision-making to pre-defined problems, whereas an organism has evolved under natural selection [182,191]. SFs are well-integrated into their ecological environment, and they show species-specific phenotypic plasticity enabling them to react to environmental changes during their lifetime. They can adapt to changes in their environment and adjust their feeding behaviour to temperature, flow velocity and particle concentration, whereas technical filters are static [69,182]. Recent developments in filtration technologies are so-called smart filters. These include filter media designed by artificial intelligence to plan tailored membranes for applications such as the selective retention of salt ions in drinking water purification [192] or surface modified filters to detect toxic polar molecules in real time [193]. Adaptive changes to the surrounding flow are active and passive in SFs. Separation media are flexible to avoid buckling or bending, in general described as reconfiguration [142]. In strong currents, the pinnules and tube feet in crinoids bend downstream, resulting in a decreased filter area, thus reducing speed-specific drag and allowing crinoids to hold their posture and continue feeding [101]. The branching patterns in gorgonians depend on ambient flow conditions, trading-off between increasing filtration area and decreasing drag force [194,195]. The baleen plates have variable porosity that changes in response to flow velocity; the higher the flow velocity the higher the porosity [175].

A limiting factor for a successful transfer is the availability of data about the SFMs, which varies strongly between the reviewed SFs. While the SFM of the blue mussel *M. edulis* is well understood, there is only one reference about the feeding mechanism in the Antarctic krill *E. superba*, despite its ecological relevance. When looking at the three main aspects, namely particles, separation medium and fluid dynamics that are involved in particle separation, it is notable that the fewest studies are on fluid dynamics, i.e. water velocity and flow regime (electronic supplementary material, table S5). It might be beneficial to first analyse the interaction of two aspects at a time, such as the interaction of particles and different separation medium materials, the influence of different geometries on fluid flow or the flow regime around different types of particles, i.e. spheres, fibres, irregular shapes.

In a biomimetic working process, we propose to focus on single traits and functions instead of transferring the complete mechanism. For example, the technology to build artificial cilia has yet to be invented, so the mucus net transported by cilia in ascidians is challenging to mimic, but the fluid flow through the pharyngeal basket might show some new insights into how changes of direction of fluids in pipes might be accomplished without high pressure losses. Progress in manufacturing processes such as additive manufacturing [196] and increasing use of numerical simulations in addition to physical models to test and verify fluid dynamics [160,164], particle encounter [151] or retention mechanisms [197] will make a technical application of SFMs more feasible in the future.

## Data accessibility

No empirical data were used in this study. All additional literature references and tables supporting this article have been uploaded as part of the electronic supplementary material.

The data are provided in electronic supplementary material [198].
